# Centrality of the periphery: Small muscle mass to bypass O_2_ transport limitations and maximize the benefits of exercise training in heart failure

**DOI:** 10.1113/EP093476

**Published:** 2026-11-15

**Authors:** Danilo Iannetta, Fabio Giuseppe Laginestra, Ryan M. Broxterman, Carla Simonelli, Markus Amann, Mara Paneroni

**Affiliations:** ^1^ Department of Clinical and Experimental Sciences University of Brescia Brescia Italy; ^2^ Department of Anesthesiology University of Utah Salt Lake City Utah USA; ^3^ Department of Internal Medicine University of Utah Salt Lake City Utah USA; ^4^ Geriatric Research, Education, and Clinical Center VAMC Salt Lake City Utah USA; ^5^ Cardio‐Respiratory Rehabilitation Unit, Istituti Clinici Scientifici Maugeri IRCSS Institute of Lumezzane Brescia Italy

**Keywords:** cardiac rehabilitation, exercise intolerance, left ventricular ejection fraction, O_2_ transport, small muscle mass exercise

## Abstract

Heart failure (HF) is a complex, systemic cardiovascular condition characterized by a severe impairment in O_2_ transport to skeletal muscle that drives exercise intolerance and reduces quality of life. Although exercise training (ET) is a cornerstone of HF clinical management, patients often exhibit limited improvements in peak oxygen uptake (${\dot{V}}_{O_{2}\mathrm{peak}}$) following traditional whole‐body, aerobic exercise recruiting a large muscle mass (e.g., cycling). In patients with HF, adaptations within skeletal muscle (e.g., capillarization) exert a proportionally greater influence on changes in ${\dot{V}}_{O_{2}\mathrm{peak}}$ than adaptations in cardiac performance. In this review, we discuss the mechanisms by which traditional ET prescription may be suboptimal to elicit peripheral adaptations and argue that dynamic exercise recruiting only a small muscle mass (e.g., quadriceps) may be more effective in achieving this goal. Indeed, small muscle mass exercise reduces the cardiovascular restraints imposed by the failing heart, allowing the muscle to reach much greater metabolic rates and to be exposed to much larger degrees of homeostatic perturbations, a key factor triggering adaptations in O_2_ transport and utilization at the peripheral level. The intent of this review is to discuss the influence of muscle mass on exercise tolerance and adaptations to ET in HF, with the ultimate goal of promoting the integration of small muscle mass exercise into contemporary cardiac rehabilitation programmes.

## Abstract (highlights)

- **What is the topic of this review?**
  This review discusses the modulatory effects exerted by the amount of active muscle mass on exercise (in)tolerance in patients with heart failure.
- **What advances does it highlight?**
  It highlights the capacity of small muscle mass exercise training to bypass the limitations on O_2_ transport to and within locomotor muscles and, thus, to potentially improve the efficacy of current cardiac‐rehabilitation programmes in heart failure.

## 1 BACKGROUND

Heart failure (HF) is a major public health concern, currently affecting ∼60 million people worldwide (Groenewegen et al., 2020) with this prevalence predicted to double by 2050 (Yan et al., 2025). Broadly defined by the inability to increase cardiac output at normal intracardiac filling pressures (Bloom et al., 2017; Borlaug, 2014), HF is often the terminal stage in the progression of multiple, often coexisting, cardiovascular and metabolic conditions including valvular incompetence, ischemic heart disease, hypertension, and diabetes. Conventionally, the spectrum of HF is categorized based on the patients’ left‐ventricular ejection fraction (LVEF): reduced (<40%; HFrEF), mildly reduced (41–49%; HFmEF) and preserved (>50%; HFpEF) LVEF. While the within‐ and between‐group pathophysiology (Khan et al., 2024), responsiveness to therapies (Inciardi et al., 2025), and disease trajectories (Lupón et al., 2018) can be remarkably different, all three phenotypes share a pronounced impairment in O_2_ transport to locomotor skeletal muscle that reduces peak oxygen uptake (${\dot{V}}_{O_{2}\mathrm{peak}}$), drives severe exercise intolerance, worsens clinical prognosis and impairs quality of life (Paneroni et al., 2021; Schmid et al., 2026; Shimiaie et al., 2015).

Although referrals to exercise training (ET)‐based cardiac rehabilitation programmes remain low (Ruano‐Ravina et al., 2016) and dependent on phenotype (Nelson et al., 2023), ET is a key non‐pharmaceutical therapy to tackle the O_2_ transport limitations and to increase ${\dot{V}}_{O_{2}\mathrm{peak}}$ in HF – the strongest predictor of mortality in this patient population (Laukkanen et al., 2022). While intuitively such improvements could be expected to result from ET‐induced enhancements in cardiac performance, the inconsistency of the changes in diastolic and systolic function (Downing & Balady, 2011; Edwards & O'Driscoll, 2022; Fukuta et al., 2019; Haykowsky et al., 2007; Pandey et al., 2015; van Tol et al., 2006) and the strong evidence that skeletal muscle adaptations (e.g., vascular control/function and mitochondrial oxidative capacity) bear a proportionally greater influence on ${\dot{V}}_{O_{2}\mathrm{peak}}$ following ET (Foulkes et al., 2024; Houstis et al., 2018) suggests that changes in exercise tolerance in HF are largely driven by peripheral adaptations.

Thus, considering the key role of skeletal muscle to influence ${\dot{V}}_{O_{2}\mathrm{peak}}$ in HF in response to ET (Erbs et al., 2010; Hambrecht et al., 1997, 1998; Haykowsky et al., 2012; Kobayashi et al., 2003; Sullivan et al., 1988; Van Craenenbroeck et al., 2010), the periphery has emerged as a ‘central’ therapeutic target for improving symptoms of exercise intolerance in patients with HF (Bunsawat et al., 2024; Paneroni et al., 2018; Tucker et al., 2018). Yet, existing ET‐based cardiac rehabilitation programmes are rarely optimized to effectively drive peripheral adaptations. Indeed, current prescriptions rely heavily on traditional, whole‐body aerobic exercises (e.g., walking, cycling), which are particularly challenging for patients with HF because they demand a large fraction of an already limited cardiac reserve. As patients may be unable to adequately meet this demand, dyspnoea and muscle fatigue precipitate rapidly even at low work rates. This can lead to premature exercise intolerance or require a reduction in exercise intensity, in a downward spiral in which the vascular and metabolic perturbations become critically low to drive local adaptations. In this context, while different mechanisms may contribute, an insufficient local adaptive stimulus may underpin the documented low efficacy of current ET strategies in increasing ${\dot{V}}_{O_{2}\mathrm{peak}}$ in HF (Bakker et al., 2018; Kemps et al., 2008; Passino et al., 2006; Rengo et al., 2020).

In contrast to aerobic whole‐body exercise, dynamic small muscle mass exercise (e.g., knee‐extension) demands a smaller fraction of the available cardiac reserve, yielding a higher downstream perfusion‐to‐muscle mass ratio (Klausen et al., 1982; Poole & Richardson, 1997). Per unit of muscle mass, this enables individuals to achieve a substantially greater ${\dot{V}}_{O_{2}\mathrm{peak}}$ (Richardson, Knight et al., 1995) and to sustain exercise at a greater percentage of that peak capacity (Iannetta et al., 2019). Small muscle mass exercise also elicits lower perceptions of dyspnoea and fatigue (Abbiss et al., 2011; MacInnis, Morris et al., 2017; Zhang et al., 2024), allowing, locally, tolerance of greater metabolic perturbations (Rossman et al., 2012, 2014; Zhang et al., 2021). Using small muscle mass exercise, studies in HF have revealed a substantial reserve in muscle perfusion and metabolic capacity, otherwise inaccessible through traditional whole‐body exercise (Esposito, Mathieu‐Costello, Shabetai et al., 2010; Magnusson et al., 1997). Using this exercise modality, training studies have reported substantial improvements in both vascular function and structure as well as mitochondrial oxidative capacity, despite the short length of their interventions (e.g., 4–6 weeks) (Esposito et al., 2011, 2018; Gustafsson et al., 2001; Hearon et al., 2022; Tyni‐Lenné et al., 1999). Importantly, these adaptations evoked significant improvements in whole‐body ${\dot{V}}_{O_{2}\mathrm{peak}}$, underscoring the capacity of small muscle mass‐based ET to not only target peripheral adaptations, but to also translate them into actual reductions in the patient's cardiovascular risk profile.

Despite the strong physiological rationale and the promising research evidence, the efficacy of dynamic small muscle mass ET in HF has remained under‐investigated in clinical research and underutilized by cardiovascular rehabilitation programmes. With the ultimate goal of emphasizing the potency of this training modality and promoting its integration within current rehabilitative strategies, the present work will (i) review the integrated mechanisms of O_2_ transport in health and the alterations associated with HF, (ii) discuss how these abnormalities can be amplified when a large amount of muscle mass is engaged during exercise, and (iii) outline research opportunities and clinical challenges related to incorporating dynamic small muscle mass exercise into existing ET‐based cardiac rehabilitation programmes. Although the benefits of ET with small muscle mass extend to other clinical populations (e.g., respiratory patients; Broxterman et al., 2021), the focus of this review is on HF.

## 2 INTEGRATED CONVECTIVE AND DIFFUSIVE MECHANISMS OF O_2_ TRANSPORT IN HEALTH

In order to support the manifold increase in O_2_ demand of skeletal muscle, the increased transfer of O_2_ from the ambient air to the mitochondria relies upon two integrated mechanisms: (i) convection within the pulmonary and circulatory systems, and (ii) diffusion across the alveolar–capillary barrier in the lungs and the capillary–mitochondrial interface in muscle. Convective O_2_ transport can be described by the Fick principle, which defines whole‐body O_2_ uptake as:

(1a) $${\dot{V}}_{O_{2}}=\dot{Q}\left(C_{{\mathrm{aO}}_{2}}-C_{\overset{-}{v}O_{2}}\right)$$

And muscle O_2_ uptake:

(1b) $${\dot{V}}_{mO_{2}}={\dot{Q}}_{m}(C_{aO_{2}}-C_{vO_{2})}$$

where $\dot{Q}$ is cardiac output, $C_{aO_{2}}$ is arterial O_2_ content, $C_{\overset{-}{v}O_{2}}$ is mixed venous O_2_ content, ${\dot{Q}}_{m}$ is muscle blood flow, and $C_{vO_{2}}$ is venous O_2_ content draining the muscle. Accordingly, $\dot{Q}$∙$C_{aO_{2}}$ and ${\dot{Q}}_{m}$∙$C_{aO_{2}}$ represent whole‐body (${\dot{Q}}_{O_{2}}$) and muscle (${\dot{Q}}_{mO_{2}}$) convective O_2_ transport, respectively. While $\dot{Q}$ and ${\dot{Q}}_{m}$ are exclusively convective factors, $C_{aO_{2}}$, $C_{\overset{-}{v}O_{2}}$ and $C_{vO_{2}}$, and therefore the arteriovenous O_2_ content difference (i.e., O_2_ extraction), are influenced by both convective and diffusive mechanisms (Wagner, 2000b). This is an important consideration because O_2_ extraction has been erroneously used to infer diffusive O_2_ transport (this misinterpretation is discussed in more detail later in this review).

In healthy individuals, $\dot{Q}$ and whole‐body ${\dot{V}}_{O_{2}}$ are tightly coupled with a typical gain of approximately 5–6 L min^−1^ of $\dot{Q}$ per 1 L min^−1^ ${\dot{V}}_{O_{2}}$ (Julius et al., 1967). The rise in $\dot{Q}$ with greater metabolic demand is met by increases in both stroke volume (at least up to ∼50% of ${\dot{V}}_{O_{2}\mathrm{peak}}$; Proctor et al., 1998; Stöhr et al., 2011) and heart rate. Stroke volume is determined by three factors: preload (ventricular end‐diastolic stretch), myocardial contractility and afterload (pressure resisting ejection) (Nixon et al., 1982; Sarnoff & Mitchell, 1961). During exercise, augmented venous return and diastolic filling increase preload, while sympathetically mediated activation of β‐adrenergic receptors on the myocardium facilitates phosphorylation of several downstream targets that increase both contraction force (positive inotropy) and speed of relaxation (positive lusitropy) (Bers, 2002), allowing the heart to eject a greater volume of blood at a given end‐diastolic volume and aortic blood pressure. Simultaneously, exercise‐induced vasodilation within the active musculature decreases systemic vascular resistance, which limits increases in afterload (Higginbotham et al., 1986). Diastolic filling occurs at relatively low filling pressures owing to compliant ventricular chambers and elastic central vasculature, permitting substantial increases in end‐diastolic volume without disproportionately raising intracardiac and pulmonary blood pressures (Gilbert & Glantz, 1989). At all intensities, but especially above ∼50% of ${\dot{V}}_{O_{2}\mathrm{peak}}$, the rise in $\dot{Q}$ is supported by an increase in heart rate which is determined by the dynamic balance between sympathetic and parasympathetic influence on the sinoatrial node (Camm & Fei, 1996). While the parasympathetic nervous system predominates at rest, during exercise the greater activity of the sympathetic branch and the withdrawal of parasympathetic activity increases the activity of the sinoatrial node and enhances the electrical conduction velocity of the heart, thus contributing to positive chronotropy (Fisher et al., 2015).

Downstream of the heart, the distribution of $\dot{Q}$ to and within skeletal muscle during exercise is the result of the dynamic adjustments necessary to maintain appropriate O_2_ provision to active muscle fibres (i.e., ${\dot{Q}}_{mO_{2}}$) and systemic blood pressure homeostasis (Joyner & Casey, 2015). This double duty is accomplished via a continuous interplay between local exercise‐induced vasodilatory compounds (e.g., nitric oxide, adenosine, prostaglandins, ATP) and sympathetically mediated systemic vasoconstrictive drive. This regulation is particularly important during large muscle mass exercise during which, due to the abundance of vasodilatory mediators and the high compliance of the skeletal muscle vascular bed, an excessive increase in vascular conductance has the potential to ‘outstrip’ $\dot{Q}$ and threaten blood pressure and perfusion to vital organs (e.g., brain) (Rowell, 1988). Therefore, a sympathetically mediated systemic vasoconstrictor drive acting on adrenergic receptors of smooth muscle is needed to counteract the potential cardiocirculatory sequela of an excessive vasodilation within contracting muscle (Rowell, 1997).

At the muscle capillary level, the diffusional movement of O_2_ from the red blood cells (RBCs) across the capillary endothelium, interstitial space, sarcolemma and myoplasm to the mitochondrion can be described by the Fick's law of diffusion:

(2) $${\dot{V}}_{O_{2}}=D_{{\mathrm{mO}}_{2}}\left(P_{\mathrm{cap}O_{2}}-P_{\mathrm{mit}{\mathrm{oO}}_{2}}\right)$$

where $D_{mO_{2}}$ represents the muscle O_2_ diffusional conductance, encompassing all impediments to the movement of O_2_ from RBCs to mitochondrion, and $P_{\mathrm{cap}O_{2}}$ and $P_{\mathrm{mit}{\mathrm{oO}}_{2}}$ are the O_2_ tensions within the muscle capillary and the mitochondrion, respectively. Because $P_{\mathrm{cap}O_{2}}$ is proportional to venous $P_{O_{2}}$ ($P_{vO_{2}}$) and $P_{\mathrm{mit}{\mathrm{oO}}_{2}}$ is close to zero (Wagner, 2023), the diffusion equation can be operationally simplified as:

(3) $${\dot{V}}_{mO_{2}}=D_{mO_{2}}kP_{vO_{2}}$$

where *k* accounts for the proportionality between $P_{\mathrm{cap}O_{2}}$ and $P_{vO_{2}}$ values. Of note, $P_{\mathrm{mit}{\mathrm{oO}}_{2}}$ has not been measured during high‐intensity exercise in patients with HF, and recent evidence indicates that $P_{\mathrm{mit}O_{2}}$ during maximal exercise is greater in untrained participants than originally reported in trained participants (Broxterman et al., 2024; Richardson, Noyszewski et al., 1995). Mean $P_{\mathrm{cap}O_{2}}$ represents the driving force for O_2_ diffusion and depends on ${\dot{Q}}_{mO_{2}}$ to the capillary and its spatial and temporal matching to ${\dot{V}}_{mO_{2}}$. $D_{mO_{2}}$ determines O_2_ flux for a given pressure gradient and depends not only on the spatial arrangement of the capillary network (e.g., capillary density, tortuosity and branching), but also on the effective capillary surface area available for O_2_ diffusion (i.e., longitudinal recruitment) (Poole et al., 2021).

The interaction between convective (Fick principle; Equation 1a and 1b) and diffusive (Fick's law of diffusion; Equations 2 and 3) mechanisms of O_2_ transport in setting the achievable ${\dot{V}}_{O_{2}}$ can be elegantly portrayed using the ‘Wagner diagram’ (Wagner, 2023) (Figure 1). Although $D_{mO_{2}}$ is most appropriately determined from the relationship between ${\dot{V}}_{mO_{2}}$ and the $P_{\mathrm{cap}O_{2}}$–$P_{\mathrm{mit}{\mathrm{oO}}_{2}}$ gradient, rather than the ${\dot{V}}_{mO_{2}}$ to $P_{vO_{2}}$ relationship, the latter likely provides a reasonable approximation of $D_{mO_{2}}$ and allows the Fick principle and Fick's law of diffusion to be illustrated on the same axes. In this diagram, the sigmoidal line illustrates the Fick principle, with the *y*‐intercept determined by convective O_2_ transport to the muscle (i.e., ${\dot{Q}}_{mO_{2}}$) and the curvilinear profile determined by the oxyhaemoglobin dissociation curve as a function of $P_{vO_{2}}$, which in turn reflects convective O_2_ transport to the capillary and its spatial and temporal matching to ${\dot{V}}_{mO_{2}}$. The straight line illustrates Fick's law of diffusion, with the slope determined by muscle O_2_ diffusional conductance (i.e., $D_{mO_{2}}$). Under the assumption of negligible $P_{\mathrm{mit}{\mathrm{oO}}_{2}}$ (Richardson, Noyszewski et al., 1995), this line originates at $P_{vO_{2}}$ equal to zero, and changes in $P_{vO_{2}}$ correspond to proportional changes in ${\dot{V}}_{mO_{2}}$ according to $D_{mO_{2}}$. Mass balance dictates that the point of the intersection between the two lines identifies the only ${\dot{V}}_{mO_{2}}$ that satisfies both equations for a given set of conditions (Figure 1). Importantly, the value of $P_{vO_{2}}$ (i.e., O_2_ extraction) is dependent on both components (as discussed in more detail later in this review). Understanding the interplay between convective and diffusive O_2_ transport is of paramount importance in patients with HF in whom the extent to which these components limit exercise tolerance may vary as a function of disease severity and phenotype (Houstis et al., 2018; Skow et al., 2024). Furthermore, as will be discussed later on in this review, active muscle mass directly influences these components, with important implications for exercise tolerance and adaptive responses to ET.

**FIGURE 1:**
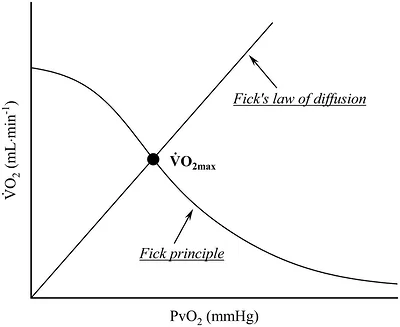
Schematic illustration of the convective and diffusive components of O_2_ transport within skeletal muscle that interact to determine the attainable ${\dot{V}}_{O_{2}\mathrm{peak}}$ during exercise. The sigmoidal lines illustrate the Fick principle, with the *y*‐intercept determined by convective O_2_ transport to the muscle (${\dot{Q}}_{mO_{2}}$) and the curvilinear profile determined by the oxyhaemoglobin dissociation curve as a function of venous partial pressure of O_2_ ($P_{vO_{2}}$), which in turn reflects convective O_2_ transport to the capillary and its spatial and temporal matching to muscle ${\dot{V}}_{O_{2}}$. The straight lines illustrate Fick's law of diffusion, with the slope determined by muscle O_2_ diffusional conductance ($D_{mO_{2}}$). The point of intersection between the two lines identifies the achievable ${\dot{V}}_{O_{2}\mathrm{peak}}$.

## 3 IMPAIRMENTS OF THE CONVECTIVE AND DIFFUSIVE MECHANISMS OF O_2_ TRANSPORT IN PATIENTS WITH HF

A hallmark feature of HF is an increase in $\dot{Q}$ that is insufficient to meet the metabolic demands of exercise (i.e., ↓ $\dot{Q}$/${\dot{V}}_{O_{2}}$ ratio) (Corrieri et al., 2021; Weber et al., 1982) and even when adequate it is nonetheless achieved at the cost of elevated cardiac filling pressures (Bhella et al., 2011). The reduced ability to raise $\dot{Q}$ is due to limitations on increasing stroke volume or heart rate (i.e., chronotropic incompetence), or both. In patients with HFrEF the impairments in raising stroke volume are largely caused by a reduced cardiomyocyte contractility and increased afterload (i.e., systolic dysfunction). In patients with HFpEF, instead, the main constraints to elevations in stroke volume are an increased myocardial stiffness and a decreased ventricular compliance (i.e., diastolic dysfunction) (Shibata et al., 2011). Despite these observations, there is increasing recognition that systolic and diastolic abnormalities often coexist across both HF phenotypes and can synergistically affect $\dot{Q}$ in response to exercise (Bloom et al., 2017; Borlaug, 2014; Pecchia et al., 2025). In addition to these stroke volume limitations, chronotropic incompetence is an important determinant of impaired $\dot{Q}$, with up to ∼80% of patients with HFrEF and HFpEF exhibiting a reduced heart rate response during exercise (Magrì et al., 2025). This reduced ability to elevate heart rate is commonly attributed to the progressive downregulation and desensitization of β‐adrenergic receptors secondary to chronic sympathetic overactivation (Zweerink et al., 2018).

The exercise‐induced increase in $\dot{Q}$ is often accompanied by a disproportionate rise in left‐sided intracardiac filling pressure, which is predominately caused by incomplete systolic emptying in HFrEF (Edward, Parker et al., 2023) and impaired myocardial relaxation and increased ventricular and arterial stiffness in HFpEF (Prasad et al., 2010). While elevated filling pressures may serve as compensatory mechanism to ensure adequacy of stroke volume, they can also contribute to lung congestion and oedema (Melenovsky et al., 2015; Merli et al., 2022; Reddy et al., 2019; Scali et al., 2017). This, in turn, compromises pulmonary gas exchange through reductions in lung diffusing capacity (Guazzi, 2008) and, possibly, greater ventilation–perfusion ($\dot{V}$/$\dot{Q}$) mismatch (Balmain et al., 2022; Woods et al., 2010). Owing to the retrograde propagation of these elevated left‐sided filling pressures (Ainab et al., 2025), up to ∼60% of patients develop right‐ventricular dysfunctions (Edward, Parker et al., 2023; Gorter et al., 2016) that lead to systemic congestion and further structural and haemodynamic abnormalities within the pulmonary system (Edward, Banchs et al., 2023; Fayyaz et al., 2018; Malhotra et al., 2016). Collectively, although an elevation in intracardiac filling pressure during exercise may help in raising $\dot{Q}$, it is at the same time considered a primary determinant of exercise intolerance in both HF phenotypes (Borlaug et al., 2010; Di Salvo et al., 1995; Shibata et al., 2011) because it is linked to ventilatory and gas exchange inefficiency and excessive dyspnoea. Notwithstanding this, recent findings have challenged the strength of this paradigm, at least in HFpEF patients, as interventions reducing pulmonary pressure did not result in improved pulmonary $\dot{V}$/$\dot{Q}$ matching nor permit the achievement of greater ${\dot{V}}_{O_{2}\mathrm{peak}}$ during incremental exercise (Balmain et al., 2023; Sarma et al., 2023).

A hallmark feature of HF is autonomic imbalance arising from abnormal activity and/or integration of one or more neurocirculatory control mechanisms (e.g., baroreflex, chemoreflex, exercise pressor reflex, central command), resulting in a net sympathetic drive that exceeds homeostatic needs (Bunsawat et al., 2024; Floras & Ponikowski, 2015; Iannetta et al., 2025). Both at rest and during exercise, HFrEF and HFpEF patients exhibit heightened muscle sympathetic nerve activity (i.e., MSNA) compared with age‐matched healthy and/or comorbid controls (Badrov et al., 2022; Bunsawat et al., 2021; Notarius & Floras, 2021; Notarius, Millar, Floras et al., 2015, 2025; Seravalle et al., 2019; Takeda et al., 2024). In addition to this exaggerated sympathetic outflow, recent in vivo studies in animals (Calvo et al., 2026) and patients (Alpenglow et al., 2023, 2024; Barrett‐O'Keefe et al., 2019) with HF have demonstrated the presence of an impaired functional sympatholysis, which has been largely attributed to a reduced bioavailability of nitric oxide (NO). Indeed, while some contrasting findings exist, several studies in both HFrEF and HFpEF patients have reported an impaired endothelium‐dependent flow‐mediated dilation of conduit arteries (Kishimoto et al., 2017; Klosinska et al., 2009) and attenuated hyperaemia to passive leg movement (Francisco et al., 2021; Witman et al., 2015) – both of which are haemodynamic responses largely mediated by NO bioavailability (Green et al., 2014; Mortensen et al., 2007; Trinity et al., 2012).

The combination of excessive sympathetic drive and reduced functional sympatholysis in patients with HF has the potential to amplify limitations to ${\dot{Q}}_{mO_{2}}$ already imposed by an insufficient, or sluggish, increase in $\dot{Q}$ during exercise. As ${\dot{Q}}_{mO_{2}}$ is a critical determinant of $P_{\mathrm{cap}O_{2}}$, a reduction in ${\dot{Q}}_{mO_{2}}$ lowers the O_2_ gradient between the capillary and the mitochondrion (Wagner, 2023). In this context, animal preparations have consistently observed a lower resting $P_{vO_{2}}$ in rat models of chronic HF and a precipitous fall to values much lower than those reported in healthy animals following the onset of muscle contraction (Behnke et al., 2007; Diederich et al., 2002). Consistent with this, several studies in patients with HFrEF and HFpEF have shown that bulk ${\dot{Q}}_{mO_{2}}$ (e.g., brachial and/or femoral artery) during exercise is reduced compared to healthy and/or comorbid controls (Amann et al., 2014; Esposito, Mathieu‐Costello, Shabetai et al., 2010; Hearon et al., 2022; Lee et al., 2016; Ratchford et al., 2020; Sullivan et al., 1989; Weavil et al., 2021). The magnitude of these reductions among studies varied from ∼15% to ∼40%, likely reflecting differences in exercise modality, pharmacological therapy, and disease severity (Wilson et al., 1984; Yamabe et al., 1995). Importantly, transient relief of sympathetically mediated vasoconstriction using acute pharmacological interventions to attenuate sympathoexcitatory pathways in patients with HFrEF (Amann et al., 2014) or to block α‐adrenergic vasoconstriction within locomotor muscles in patients with either HFrEF or HFpEF (Alpenglow et al., 2024; Barrett‐O'Keefe et al., 2019) increased ${\dot{Q}}_{mO_{2}}$ during exercise, the magnitude of which was markedly greater than that observed in matched healthy controls. Moreover, augmenting NO bioavailability has been documented to improve exercise tolerance in HFpEF (Borlaug et al., 2015; Zamani et al., 2015) and to speed the ${\dot{V}}_{O_{2}}$ kinetics in HFrEF (Sperandio et al., 2012), likely via mechanisms related to an increased ${\dot{Q}}_{mO_{2}}$ and/or better spatial and temporal matching with ${\dot{V}}_{mO_{2}}$ (Craig et al., 2019; Ferguson et al., 2016; Glean et al., 2015). Collectively, these in vivo investigations provide strong evidence for the overactivity of the sympathetic branch of the autonomic system and the inadequacy of the peripheral vasculature to oppose it as key mechanisms impairing convective O_2_ transport to the active muscle across the spectrum of HF.

In addition to abnormalities in convective O_2_ transport to skeletal muscle, HF patients exhibit substantial structural remodelling of the microvascular network which has been linked to a more general remodelling of skeletal muscle (Paneroni et al., 2018). Both HFrEF and HFpEF patients manifest marked reductions in capillary density and capillary‐to‐fibre ratio in parallel with a significant fibre type shift directionally from more oxidative and more fatigue‐resistant type I muscle fibres to more glycolytic, less fatigue‐resistant type II (Kitzman et al., 2014; Magnusson, Kaijser et al., 1996; Sullivan et al., 1990; Williams et al., 2004). The result of this capillary rarefaction is an increased heterogeneity between ${\dot{Q}}_{mO_{2}}$ and ${\dot{V}}_{mO_{2}}$ (Kissane et al., 2021) which can reduce O_2_ diffusional conductance from the vascular bed to the mitochondria (Wagner, 2000a). In addition to this, a factor that likely bears an even greater impact to the diffusion of O_2_ is the observation that the proportion of capillaries supporting RBC flux both at rest and during muscle contraction is reduced in HF (Kindig et al., 1999; Richardson et al., 2003). Because the number of capillaries – and capillary length – supporting RBC flux is a critical determinant of $D_{mO_{2}}$ (Poole & Musch, 2023), the impaired ability to adequately elevate capillary haematocrit is deemed to be a key factor for the reduced exercise tolerance of patients with HF (Poole et al., 2012). Consistent with these observations, a number of studies combining arterial and venous catheterization alongside measurements of conduit artery blood flow has revealed substantial reductions in $D_{mO_{2}}$ (i.e., −20% to −30%) during exercise in both HFrEF and HFpEF patients (Esposito, Mathieu‐Costello, Shabetai et al., 2010; Houstis et al., 2018) with the magnitude of this reduction strongly predictive of impairments in ${\dot{V}}_{O_{2}\mathrm{peak}}$. Interestingly, HFpEF patients have been documented to possess a relatively lower $D_{mO_{2}}$ compared to age‐matched HFrEF patients, suggesting that capillary haemodynamics may be even poorer in HF patients with an apparently normal LVEF (Dhakal et al., 2015).

It is important to note that the arteriovenous O_2_ difference measured at maximal exercise across the spectrum of LVEFs has been reported to be normal or even enhanced compared to healthy controls (Abudiab et al., 2013; Esposito, Mathieu‐Costello, Shabetai et al., 2010; Kitzman et al., 1991; LeJemtel et al., 1986; Martin et al., 1989; Parovic et al., 2019; Pugliese et al., 2021; Reddy et al., 2018; Santos et al., 2015; Sullivan et al., 1989). These findings seem to contrast with the aforementioned reduction in O_2_ diffusive conductance and have, indeed, been used to support the perilous misconception that the periphery may not be a meaningful contributor to the poor exercise tolerance of patients with HF. However, proper interpretation of the relationship between muscle perfusive and diffusive components of fractional O_2_ extraction permits reconciling these seemingly contrasting findings. Specifically:

(4) $$O_{2}\text{extraction}={\dot{V}}_{O_{2}}/\;{\dot{Q}}_{m}={\dot{Q}}_{m}\left[1-exp\left(-D_{mO_{2}}/\beta {\dot{Q}}_{m}\right)\right]$$

Because fractional O_2_ extraction is largely determined by the ratio between $D_{mO_{2}}$ and ${\dot{Q}}_{m}$, a reduction in ${\dot{Q}}_{m}$ increases the time available for O_2_ unloading, thereby allowing fractional O_2_ extraction to increase even in the presence of a reduced $D_{mO_{2}}$. Thus, with progressively lower levels of $\dot{Q}$ and ${\dot{Q}}_{mO_{2}}$, O_2_ extraction will tend to progressively increase (Wilson et al., 1984). Consequently, a normal or even enhanced fractional O_2_ extraction in patients with HF should not be interpreted as evidence that skeletal muscle O_2_ diffusive conductance is preserved or that peripheral limitations to O_2_ transport do not contribute to the impaired exercise tolerance. Rather, a high fractional O_2_ extraction may coexist with impaired O_2_ diffusional conductance when ${\dot{Q}}_{mO_{2}}$ is critically reduced. By the same token, a lower‐than‐normal fractional O_2_ extraction is not necessarily synonymous with an impaired ability to extract O_2_ by mitochondria.

## 4 A LARGER ACTIVE MUSCLE MASS EXACERBATES O_2_ TRANSPORT LIMITATIONS AND WORSENS EXERCISE INTOLERANCE IN PATIENTS WITH HF

As outlined above, patients with either HFrEF or HFpEF exhibit impairments at multiple levels of the O_2_ transport cascade that collectively predispose them to premature fatigue and exercise intolerance. During exercise involving a large muscle mass (e.g., walking, cycling), these impairments are likely to become more pronounced because the greater active muscle mass demands a large fraction of the limited cardiac reserve accentuating the functional consequences of the cardiovascular, pulmonary, autonomic and perceptual abnormalities associated with HF (Figure 2a). Below we describe six major mechanisms that could contribute to reduce exercise tolerance when engaging a large amount of muscle mass and impact the ability to accumulate sufficient doses of ET. It is important to note that these mechanisms could all be present within a given patient and interact with each other. It is also key to consider that evidence supporting the influence of some of these mechanisms is available only for healthy individuals, and therefore, their involvement in HF remains a working hypothesis.

**FIGURE 2:**
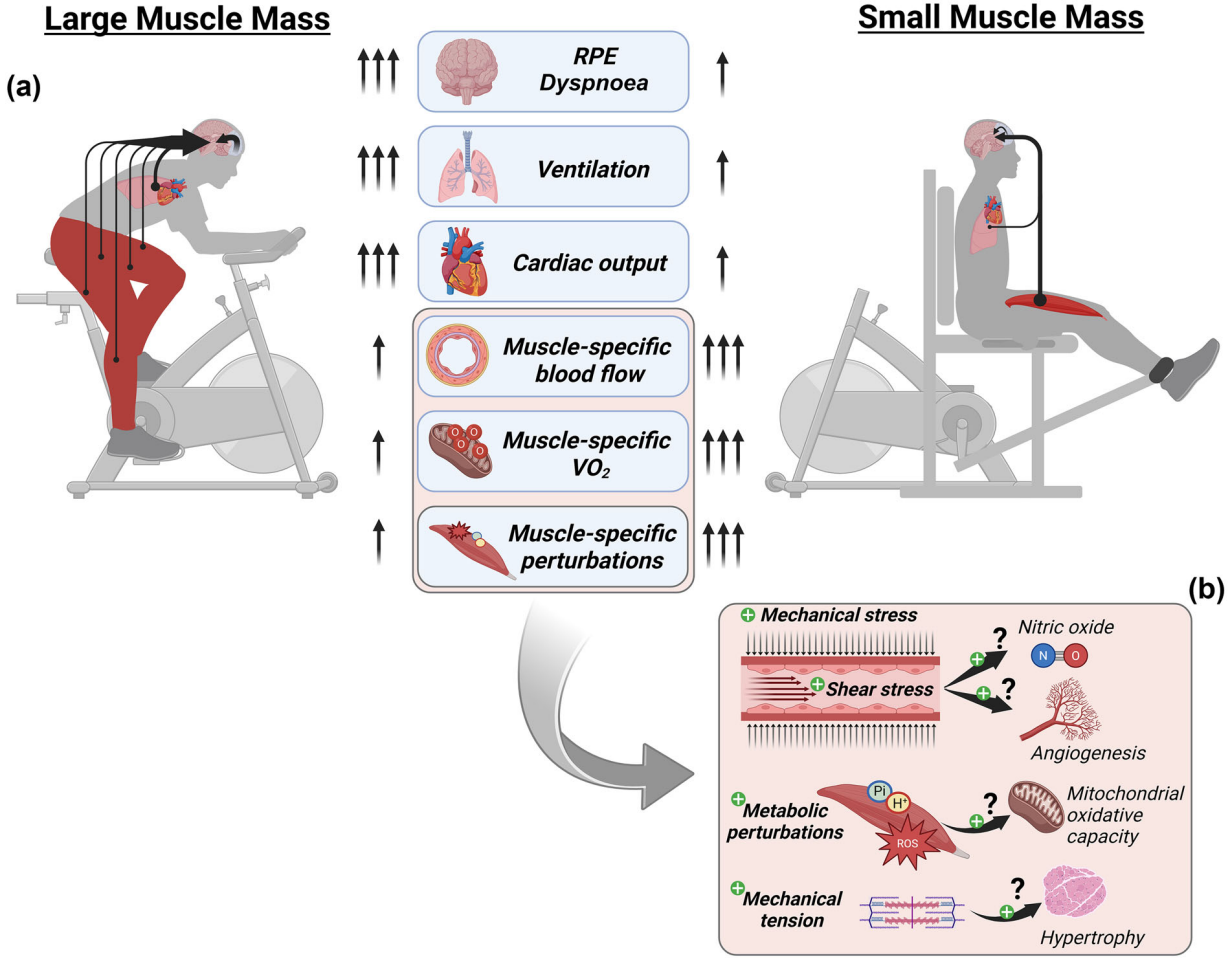
(a) Schematic illustration of how active muscle mass may modulate perceptual, respiratory, cardiocirculatory and metabolic responses during exercise. For the same muscle‐specific metabolic rate, exercise involving a large muscle mass (e.g., cycling) requires greater ventilatory and cardiac output responses than exercise involving a small muscle mass (e.g., knee‐extension). Furthermore, the greater work of breathing, together with increased central motor drive to and afferent feedback from the working muscles is associated with a higher perception of effort and dyspnoea than during small muscle mass exercise. At the peripheral level, small muscle mass exercise permits: (i) greater O_2_ availability per unit of muscle mass, (ii) a higher muscle‐specific metabolic rate, and (iii) tolerance of greater metabolic perturbation. (b) The working hypothesis is that these small muscle mass‐related dynamics provide a stronger stimulus compared to large muscle mass exercise for improving vascular function, promoting angiogenesis and mitochondrial biogenesis, and facilitating hypertrophy.

### 4.1 Increased intracardiac filling pressure

For a given increase in $\dot{Q}$, patients with HF experience an approximately three‐fold greater rise in intracardiac filling pressures than healthy controls (Chatterjee et al., 2013; Edward, Parker et al., 2023; Eisman et al., 2018; Reddy et al., 2018). Therefore, for the same muscle‐specific metabolic rate, adding muscle mass to exercise will be associated with a disproportionate elevation in intracardiac filling pressures, increasing the likelihood of developing pulmonary congestion and oedema, which would then impair pulmonary O_2_ exchange and promote arterial hypoxaemia (Leahy et al., 2025).

### 4.2 Greater work of breathing and hierarchical redistribution of blood flow

Large muscle mass exercise is associated with an inherently greater work of breathing compared to small muscle mass exercise. Based on the hierarchical distribution of blood flow, with progressively higher levels of work of breathing, the respiratory muscles demand an increasingly larger portion of the available $\dot{Q}$ (Dominelli et al., 2017; Harms et al., 1997). In HF, mechanical constraints, ongoing ventilatory inefficiencies, and greater chemoreflex stimulation from arterial hypoxaemia (Lalande et al., 2020) can accentuate the work of breathing and activate the so‐called ‘respiratory muscle metaboreflex’ which augments sympathetic outflow disproportionally (Hill, 2000), causing a greater portion of blood flow to be redirected away from exercising muscles to respiratory muscles (Olson et al., 2010). In an animal model of pulmonary hypertension (a condition common to many HF patients), the extent to which ${\dot{V}}_{O_{2}\mathrm{peak}}$ was reduced compared to controls was proportional to the extent to which diaphragm ${\dot{Q}}_{mO_{2}}$ was raised during exercise (Schulze et al., 2025), suggesting a strong relationship in HF between the blood flow ‘stealing’ phenomenon from respiratory muscles and reductions in exercise tolerance.

### 4.3 Excess sympathoexcitatory drive

In healthy individuals, recordings of MSNA demonstrate that sympathetic activity is suppressed for mild‐to‐moderate intensities during one‐leg cycling exercise, whereas in both HFrEF and HFpEF patients nerve traffic is augmented, with the magnitude of this increase being associated with the reduction in ${\dot{V}}_{O_{2}\mathrm{peak}}$ (Badrov et al., 2022; Notarius, Millar, Murai et al., 2015, Notarius et al., 2025). Abnormal inputs and/or integration of feedback from chemoreceptors and/or group III/IV mechano‐ and metabo‐sensitive afferents, together with impaired baroreceptor function, may contribute to this excess sympathetic activity (Iannetta et al., 2025; Keir et al., 2020; Smith et al., 2023). Given that sympathetic outflow may increase with the amount of active muscle mass, particularly at higher exercise intensities (Katayama & Saito, 2019), it is plausible that whole‐body exercise may further amplify this response, resulting in an even greater vasoconstrictor influence compared with small muscle mass exercise and further reduction of ${\dot{Q}}_{mO_{2}}$.

### 4.4 Further slowing of ${\dot{V}}_{O_{2}}$ kinetics and faster development of muscle fatigue

The excess sympathetic activity and the blood flow ‘stealing’ phenomenon from respiratory muscle could exacerbate the already impaired ${\dot{Q}}_{mO_{2}}$ and $P_{\mathrm{cap}O_{2}}$ from the onset of muscle contractions (Poole et al., 2012). This could substantially slow ${\dot{V}}_{mO_{2}}$ kinetics and enlarge the O_2_ deficit (Chatterjee et al., 2013; Hearon et al., 2019) promoting greater reliance on glycolytic sources of energy early on during the exercise (Richardson et al., 1998). This, in turn, accelerates metabolite accumulation and the consequent development of muscle contractile impairment (Weavil et al., 2021; Weiss et al., 2017).

### 4.5 Increased ensemble activity of group III/IV muscle afferents

While at (near‐)maximal exercise intensity the intramuscular metabolic milieu is more disturbed with smaller active muscle mass (Rossman et al., 2012, 2014), the ensemble feedback from group III/IV afferents is overall greater during large muscle mass exercise because it originates from more muscle groups and is supplemented by greater signals from respiratory muscles and the heart (Thomas et al., 2018). While the evidence in HF is scarce at the moment, it is well established that greater ensemble feedback from group III/IV muscle afferents during large muscle mass can exert inhibitory action on descending motoneuronal drive (Blain et al., 2016; Sidhu et al., 2018), critically limiting the capacity to sustain exercise.

### 4.6 Altered perceptual status

A larger amount of active muscle mass requires a proportionally higher central motor drive (Franke et al., 2000). In HF this requirement may be increased more rapidly in order to compensate for the rapid fatigue development of the recruited motor units (Weavil et al., 2021). Although speculative in HF, the accentuated central motor drive and the greater ensemble feedback from group III/IV muscle afferents (see previous point) can exacerbate perception of effort leading to a more rapid achievement of exercise intolerance (Weavil & Amann, 2019).

## 5 QUANTIFYING DIFFERENCES IN CONVECTIVE AND DIFFUSIVE MECHANISMS OF O_2_ TRANSPORT BETWEEN LARGE VS. SMALL MUSCLE MASS EXERCISE IN HF

From our discussion thus far, larger amounts of active muscle mass challenge the already compromised adequacy of O_2_ availability within skeletal muscle to a greater extent, critically affecting the power‐generating capacity of the muscle group engaged (Figure 2a). To quantify the differences in O_2_ transport to locomotor muscle between larger and smaller amount of exercising muscle mass, we reconstructed the Wagner diagram from Esposito and colleagues (Esposito, Mathieu‐Costello, Shabetai et al., 2010) who compared the muscle convective and diffusive O_2_ transport determinants to ${\dot{V}}_{mO_{2}}$ in catheterized patients with HFrEF during bipedal cycling and knee‐extension exercise. We plotted the mean data for the Fick principle and Fick's law of diffusion, normalizing ${\dot{V}}_{mO_{2}}$ to the leg‐muscle mass involved in each exercise modality (Figure 3). Per 100 g of active muscle mass, ${\dot{Q}}_{mO_{2}}$ and $D_{mO_{2}}$ were approximately 100% greater during knee‐extension exercise than during cycling. This observation is noteworthy because it indicates that, in HF, knee‐extensor exercise improves both the convective and the diffusive determinants of muscle O_2_ transport relative to cycling, thereby creating a more favourable condition for a higher attainable ${\dot{V}}_{mO_{2}\mathrm{peak}}$. Indeed, the combined increase in convective and diffusive O_2_ transport capacities approximately doubled ${\dot{V}}_{mO_{2}\mathrm{peak}}$ per unit of muscle mass, revealing a substantial muscle metabolic reserve in these patients with HF (Figure 3). Relative to healthy controls, switching from large to small muscle mass exercise reduced the impairment in ${\dot{Q}}_{mO_{2}}$ per unit of muscle mass in the patients with HF from ∼50% during cycling to ∼25% during knee‐extension exercise (Esposito et al., 2011). These findings underscore the magnitude of the restraint on ${\dot{Q}}_{mO_{2}}$ during whole‐body exercise in HF and suggest that engaging smaller amounts of active muscle mass increases both convective and diffusive components of O_2_ transport to skeletal muscle in HF.

**FIGURE 3:**
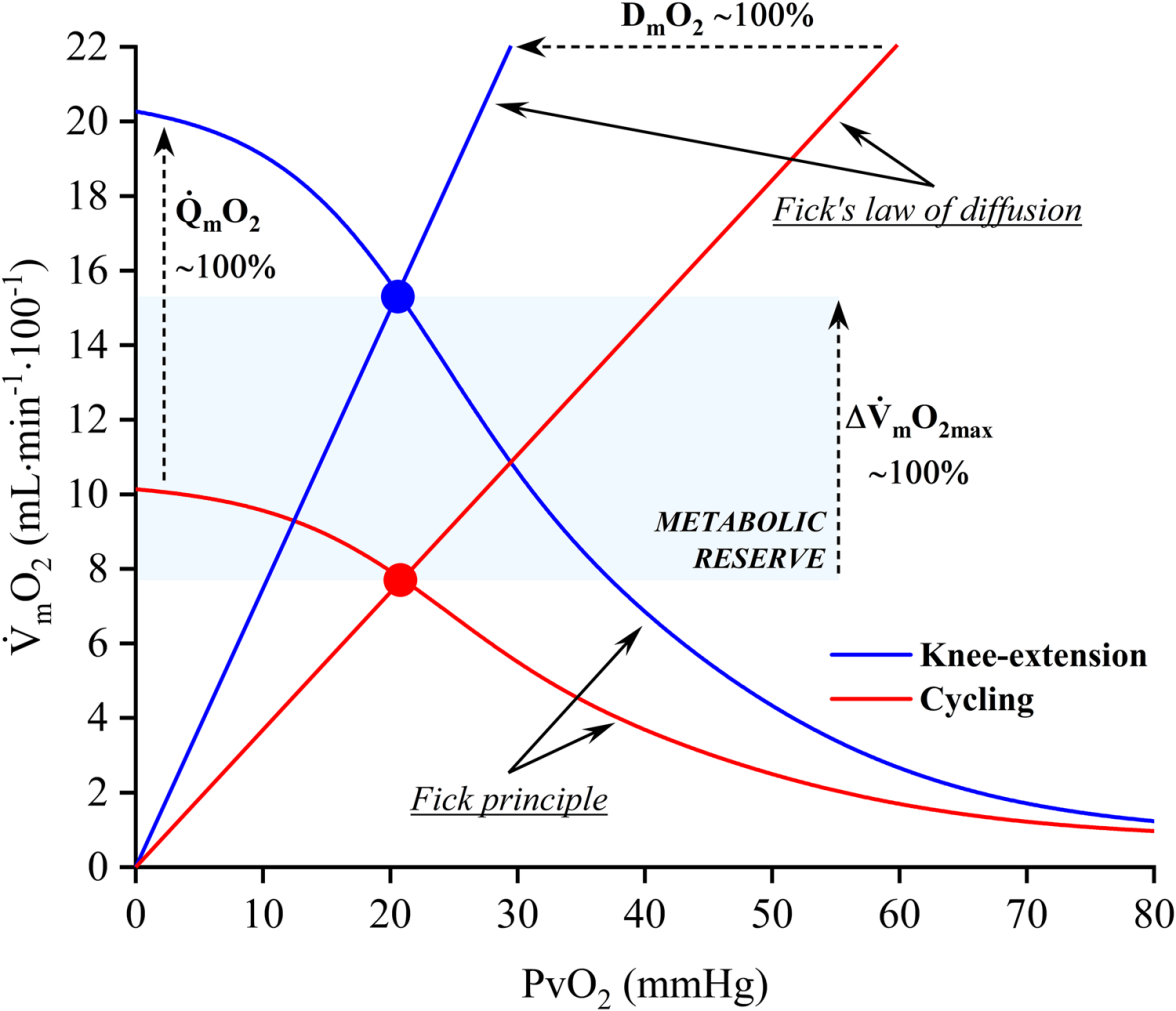
Schematic illustration of the convective and diffusive components of O_2_ transport within skeletal muscle that interact to determine the attainable muscle ${\dot{V}}_{O_{2}\mathrm{peak}}$ (${\dot{V}}_{mO_{2}\mathrm{peak}}$) during cycling and knee‐extension (KE) exercise in patients with HFrEF. The data used to model these relationships were derived from the mean values reported in Table 2 of Esposito et al. (Esposito, Mathieu‐Costello, Shabetai et al., 2010). ${\dot{V}}_{mO_{2}}$ values were normalized to the amount of muscle mass involved in each exercise modality. Per 100 g of active muscle mass, ${\dot{Q}}_{mO_{2}}$ and $D_{mO_{2}}$ were approximately 100% greater during knee‐extension exercise (small muscle mass) than during cycling (large muscle mass). Consequently, the combined increase in convective and diffusive O_2_ transport capacities approximately doubled the attainable ${\dot{V}}_{mO_{2}\max}$ per unit of muscle mass, revealing a substantial skeletal muscle metabolic reserve in these patients with HFrEF (Δ${\dot{V}}_{mO_{2}}$).

## 6 A SMALLER ACTIVE MUSCLE MASS TO TARGET PERIPHERAL O_2_ TRANSPORT LIMITATIONS AND ENHANCE CARDIORESPIRATORY FITNESS OF PATIENTS WITH HF

The documented improvements in ${\dot{V}}_{O_{2}\mathrm{peak}}$ following ET in patients with HF are largely driven by adaptations occurring at the skeletal muscle level rather than within the heart. Indeed, the responsiveness of both systolic and diastolic cardiac function to ET documented by the current literature remains highly inconsistent (Downing & Balady, 2011; Edwards & O'Driscoll, 2022; Fukuta et al., 2019; Haykowsky et al., 2007; Pandey et al., 2015; van Tol et al., 2006). On the other hand, the periphery has demonstrated marked plasticity in vascular control, vascular remodelling and mitochondrial oxidative capacity (Erbs et al., 2010; Hambrecht et al., 1997, 1998; Haykowsky et al., 2012; Katz et al., 1997; Kobayashi et al., 2003; Sullivan et al., 1988; Van Craenenbroeck et al., 2010). In addition to this evidence, empirical modelling in patients with HFpEF further indicates that peripheral adaptations of O_2_ transport exert a proportionally greater impact on ${\dot{V}}_{O_{2}\mathrm{peak}}$ than would plausible cardiac adaptations in response to ET (i.e., increase in $\dot{Q}$) (Houstis et al., 2018). Taken together, these findings emphasize the centrality of the periphery as a key therapeutic target for increasing ${\dot{V}}_{O_{2}\mathrm{peak}}$ in HF.

Amongst the various components of ET, exercise intensity plays a pivotal role in determining the magnitude of peripheral adaptations (Bi et al., 2026; MacInnis & Gibala, 2017). For instance, endothelial shear stress increases in proportion to exercise intensity (Green et al., 2005), and repeated exposure to high shear stress activates molecular pathways that enhance NO bioavailability and stimulate angiogenic growth factors (such as vascular endothelial growth factor (VEGF)) (Gustafsson et al., 1999; Wragg et al., 2014). Furthermore, higher exercise intensities impose greater mechanical compression on the endothelium, activating mechano‐transductive pathways that further support angiogenesis (Hoier & Hellsten, 2014). With respect to mitochondrial oxidative capacity, although some debate exists (Bishop et al., 2025), exercise intensity is widely regarded as a key determinant of mitochondrial adaptations (MacInnis, Zacharewicz et al., 2017). High‐intensity exercise has been shown to rapidly upregulate enzymes involved in oxidative phosphorylation (Perry et al., 2010), whereas low‐intensity exercise may not be as effective (MacInnis & Gibala, 2017).

While possible (Wisløff et al., 2007), ET programmes including high‐intensity whole‐body exercise are challenging in patients with HF because of the aggravation of the mechanisms of exercise intolerance previously discussed – not to mention that higher exercise intensities may be unfeasible due to safety concerns (Arena et al., 2013). Therefore, in order to accumulate a sufficient volume of exercise, ET intensity is often prescribed within the lower range of a patient's intensity spectrum (Hansen et al., 2023), even when the original goal of the prescription is to evoke a ‘high’ level of metabolic disturbance (Ellingsen et al., 2017). As can be inferred from the study of Esposito et al. (Esposito, Mathieu‐Costello, Shabetai et al., 2010), whole‐body high‐intensity exercise prescriptions in patients with HF leave a larger proportion of the available vascular and metabolic reserve unexploited than in age‐matched healthy controls (Figure 3), ultimately imposing a substantially weaker peripheral adaptive stimulus.

By freeing the muscle from the cardiovascular, pulmonary, autonomic and perceptual consequences associated with the failing heart, small muscle mass exercise permits tolerance of a much higher local metabolic intensity. This, in turn, exposes the muscle to a wider range of vascular and metabolic fluctuations that are critical for inducing local adaptations (Bi et al., 2026; Liu et al., 2022). For instance, during small muscle mass exercise, the combination of greater ${\dot{Q}}_{mO_{2}}$ and endothelial compression likely exposes the local vasculature to much higher shear and mechanical stresses, providing a potent stimulus for NO production and for angiogenesis (Gustafsson et al., 1999). In this context, acute bouts of unilateral knee‐extension exercise have been demonstrated to substantially increase VEGF expression in the skeletal muscle of patients with HF, although direct comparisons with large muscle mass exercise are not available (Esposito, Mathieu‐Costello, Entin et al., 2010; Gustafsson et al., 2001). Moreover, the greater metabolic perturbations achievable at (near‐)maximal exercise intensities, as evidenced by a greater muscle fatigue following small vs. large muscle mass exercise (Zhang et al., 2025), likely provide a stronger stimulus for mitochondrial biogenesis (Abbiss et al., 2011; Layec et al., 2018; Norrbom et al., 2004). The greater muscular tension that can be sustained during small muscle mass exercise may also provide an important stimulus for muscle hypertrophy (Esposito et al., 2011; Magnusson, Gordon et al., 1996), which is less likely to occur with whole‐body aerobic exercise (Grgic et al., 2019). Finally, because the perceptions of dyspnoea, fatigue and effort are generally lower with small muscle mass exercise (Abbiss et al., 2011; MacInnis, Morris et al., 2017; Zhang et al., 2024), a greater exercise volume may be accumulated, thereby enhancing the overall peripheral adaptive stimulus (Granata et al., 2018; Liu et al., 2022).

## 7 CURRENT EVIDENCE SUPPORTING THE INCORPORATION OF DYNAMIC SMALL MUSCLE MASS ET WITHIN CONTEMPORARY CARDIAC REHABILITATION PROGRAMMES TARGETING PATIENTS WITH HF

Despite the strong physiological rationale, only a handful of studies have evaluated the efficacy of dynamic small muscle mass ET in HF patients, yet all have reported significant improvements in whole‐body ${\dot{V}}_{O_{2}\mathrm{peak}}$ (Table 1). In two reports derived from the same dataset, Gordon et al. and Tynni‐Lenné et al. showed that unilateral knee‐extension ET was more effective than cycling ET in patients with HFrEF, resulting in greater improvements in both cycling peak work rate (+26 vs. + 11 W) and ${\dot{V}}_{O_{2}}$ (+3.0 vs. + 0.3 mL/kg/min) (Gordon et al., 1999; Tyni‐Lenné et al., 1999). From the series of studies of Esposito and colleagues, unilateral knee‐extension exercise led to comparable improvements in cycling peak work rate (+26 W) and ${\dot{V}}_{O_{2}}$ (+3.3 mL/kg/min) in patients with HFrEF. These improvements were largely attributed to increased quadriceps capillarization and a shift in fibre‐type expression, from more glycolytic type II fibres towards more oxidative type I fibres – changes that facilitated the reported increases in ${\dot{Q}}_{mO_{2}}$ and $D_{mO_{2}}$ (Esposito et al., 2011). More recently, Munch et al. (2018) reported a 9 W and a 2.0 mL/kg/min increase in cycling peak work rate and ${\dot{V}}_{O_{2}}$, respectively, following unilateral cycling training in patients with mildly reduced LVEF. Similarly, Hearon et al. (2022) documented increases in both cycling peak work rate (+11 W) and ${\dot{V}}_{O_{2}}$ (+1.6 mL/kg/min) following unilateral knee‐extension ET in patients with HFpEF. Interestingly, these two latter studies also reported improvements in vasomotor regulation following ET, as evidenced by an increased sympatholytic capacity (Munch et al., 2018) and greater vascular conductance and femoral blood flow in response to exercise (Hearon et al., 2022).

**TABLE 1:** Effects of dynamic small muscle mass exercise training on cycling ${\dot{V}}_{O_{2}\mathrm{peak}}$ in patients with HF.

Authors	HF phenotype	*n*	LVEF	Exercise modality	Exercise training characteristics				Δ ${\dot{V}}_{O_{2}\mathrm{peak}}$
					Total duration (weeks)	Frequency (sessions/week)	Session duration	Intensity	
Gordon et al. (1999)^*^, and Tyni‐Lenné et al. (1999)^*^	HFrEF	8	31 ± 9%	Unilateral dynamic knee‐extension	8	3	∼17 min/leg	50–70% of knee‐extension peak work rate	**↑**
	HFrEF	8	29 ± 13%	Bilateral cycling exercise	8	3	20 min	50–70% of cycling peak work rate	**↔**
Esposito et al. (2011)	HFrEF	6	25 ± 3%	Unilateral dynamic knee‐extension	8	3	50 min/leg	Various (moderate continuous, high‐intensity intervals)	**↑**
Munch et al. (2018)	HFrEF	8	45 ± 5%	Unilateral cycling	6	3	∼20 min/leg	4 × 4 min/leg intervals at ∼90% of single‐leg peak work rate	**↑**
Hearon et al. (2022)	HFpEF	8	n/a	Unilateral dynamic knee‐extension	8	3	30 min/leg	Various (i.e., moderate continuous, high‐intensity intervals)	**↑**

* These two studies report findings from the same data acquisition and thus were merged together.

Despite considerable differences in average session duration, intervention length and exercise pattern (Table 1), the magnitude of the improvements in exercise capacity and ${\dot{V}}_{O_{2}\mathrm{peak}}$ documented by these studies is remarkably consistent. This is in contrast with the marked variability of the changes in exercise capacity reported following routine cardiac‐rehabilitation programmes (Bakker et al., 2018; Rengo et al., 2020) as well as randomized controlled trials (Ellingsen et al., 2017), with up to ∼50% of patients across the spectrum of HF showing no clinically meaningful improvements in ${\dot{V}}_{O_{2}\mathrm{peak}}$ (Bakker et al., 2018; Kitzman et al., 2016; Rengo et al., 2020). Although no randomized‐controlled trial has systematically compared the effectiveness of small vs. large muscle mass ET in HF patients, the consistency of the outcomes reported by the current literature suggests that small muscle mass ET may indeed provide a potent and reliable stimulus for the molecular signalling processes underlying peripheral adaptations – a feature that may be more difficult to obtain in HF from traditional whole‐body aerobic ET.

## 8 OPPORTUNITIES, BENEFITS AND CHALLENGES OF DYNAMIC SMALL MUSCLE MASS ET FOR PATIENTS WITH HF

Although basic cardiovascular physiology and the available, albeit limited, training studies provide a strong rationale for incorporating small muscle mass ET into current cardiac rehabilitation programmes for patients with HF, adequately powered studies are needed to establish its comparative effectiveness. These studies should examine the pre–post effectiveness of small muscle mass vs. large muscle mass ET on ${\dot{V}}_{O_{2}\mathrm{peak}}$ and determine whether the effectiveness of small muscle mass ET is consistent across HF phenotypes. Indeed, although reductions in ${\dot{V}}_{O_{2}\mathrm{peak}}$ are ubiquitous across the spectrum of LVEFs (Schmid et al., 2026), the haemodynamic mechanisms underlying this impairment may differ between HF phenotypes. Reductions in $\dot{Q}$ appear to contribute more with progressively lower levels of LVEFs (Pugliese et al., 2019), while reductions in O_2_ diffusion conductance are deemed to exert a greater influence when LVEF is preserved (Dhakal et al., 2015). As such the extent and the mechanisms by which small muscle mass ET would benefit patients could also vary. In connection with these aspects, future studies should also evaluate whether factors such as baseline cardiorespiratory fitness, age, biological sex, pharmacological therapy and presence of comorbidities affect the magnitude of the adaptations (Figure 2b). Once the comparative effectiveness of small vs. large muscle mass ET has been established over longer interventional periods (e.g., >12 weeks), future research should investigate how small muscle mass ET can be optimally integrated into existing cardiac rehabilitation programmes, particularly considering that training one muscular group at a time (e.g., quadriceps) leads to a doubling of the training session duration. In this context, a careful examination of the exercise prescription strategies reported in the current literature suggests that lower‐intensity, continuous protocols and higher‐intensity, intermittent protocols produce similar gains (Table 1). Unlike whole‐body exercise (Inglis et al., 2024), lower intensities during small muscle mass ET may already exceed the peripheral stimulus threshold required to elicit adaptations. Nevertheless, as training progresses, higher‐intensity exercise may become necessary to provide an adequate stimulus for continued improvement. Similarly, cross‐study comparisons indicate that exercise duration does not appear to have a substantial influence on the observed adaptations, with programmes as short as 20 min/leg being similarly effective as those lasting 50 min/leg (Table 1). If confirmed by future studies, this latter observation is noteworthy because it would support a time‐efficient use of small muscle mass ET. It is also important to consider, though, that exclusive reliance on small muscle mass ET may become insufficient to sustain long‐term adaptations and/or to address the broader functional demands of activities of daily living. A combined approach incorporating both small and large muscle mass ET may represent the most effective rehabilitation strategy. Within such a framework, small muscle mass ET could be implemented during the early stages of cardiac rehabilitation to rapidly improve functional capacity and prepare patients for a gradual transition towards traditional whole‐body exercise.

Addressing these research questions will be essential to support the integration of small muscle mass ET into contemporary cardiac rehabilitation programmes – an integration that will, nevertheless, face several practical challenges. One of these is the lack of user‐friendly dynamic small muscle mass ergometers currently available for clinical use. Although several research groups have developed customized solutions, such as knee‐extension ergometers and cycle ergometers with counterweights to enable single‐leg cycling, these are affected by practical and technical constraints that limit their applicability. For instance, dynamic knee‐extension ergometers are bulky, require frequent calibration, and their resistance is manually controlled, which is impractical for clinical centres and not ideal for ensuring precision and reproducibility. On the other hand, single‐leg cycling configurations are poorly suited to the predominantly older and frequently overweight HF population. As such, the widespread implementation of small‐muscle mass ET in cardiac rehabilitation will have to await the development of dedicated ergometers that are cost‐effective, user‐friendly and readily integrated into existing clinical workflows.

Overcoming these barriers could substantially improve risk management for patients. Evidence suggests that each 1 mL/kg/min increase in ${\dot{V}}_{O_{2}\mathrm{peak}}$ is associated with an approximately 15% reduction in cardiovascular risk of mortality in HF (Corrà et al., 2013; Prokopidis et al., 2025). In this context, the available findings consistently support the ability of small muscle mass ET to improve ${\dot{V}}_{O_{2}\mathrm{peak}}$ in patients with HF (Table 1). Beyond its physiological benefits, this training modality could also contribute to enhance participation and adherence to cardiac rehabilitation. Fear of exercise, particularly concerns about recurrent cardiac events, represents an important barrier to participation among patients with HF (Hoffmann et al., 2018). The substantially lower cardiovascular strain associated with small muscle mass ET could augment the perception of safety during exercise and, therefore, the willingness to participate. Moreover, perceived benefits are also an important determinant of adherence to cardiac rehabilitation (Nelson et al., 2022). By eliciting substantial improvements within a relatively short timeframe, small‐muscle ET is well suited to combat programme withdrawal, which normally occurs early in the rehabilitative process (Wittmer et al., 2012).

## 9 CONCLUSIONS

Amongst patients with cardiovascular disease undergoing ET‐based cardiac rehabilitation, those with HF exhibit some of the lowest adaptive responsiveness (Bakker et al., 2018; Rengo et al., 2020). In this review, we contend that a major reason for this limited responsiveness is the inability of the traditional, whole‐body exercise prescription used by the majority of the programmes to effectively stimulate peripheral adaptations in locomotor muscle O_2_ transport and utilization – key mechanisms underlying improvements in ${\dot{V}}_{O_{2}\mathrm{peak}}$ in patients with HF (Foulkes et al., 2024; Houstis et al., 2018). We further contend that these mechanisms are more effectively stimulated through aerobic, dynamic small muscle mass ET. Indeed, by permitting fuller exploitation of the muscle's capacity to produce work, this modality exposes the working muscle to a much wider range of vascular and metabolic fluctuations and thereby enhances the stimulus for peripheral adaptations. Although direct comparative evidence is limited at the moment, the available findings provide a strong rationale for incorporating small muscle mass ET into clinical rehabilitation strategies for patients with HF with the aim of maximizing the improvements in cardiorespiratory fitness and quality of life.

## AUTHOR CONTRIBUTIONS

Danilo Iannetta: conceptualization, writing original draft, editing. Fabio Giuseppe Laginestra: conceptualization, writing original draft, editing. Ryan M. Broxterman: conceptualization, writing original draft, editing. Carla Simonelli: conceptualization, writing original draft, editing. Markus Amann: conceptualization, writing original draft, editing. Mara Paneroni. conceptualization, writing original draft, editing. All authors have read and approved the final version of this manuscript and agree to be accountable for all aspects of the work in ensuring that questions related to the accuracy or integrity of any part of the work are appropriately investigated and resolved. All persons designated as authors qualify for authorship, and all those who qualify for authorship are listed.

## CONFLICT OF INTEREST

None declared.

## GENERATIVE AI STATEMENT

No generative AI tools were used in the preparation of this manuscript.
